# Relationship between the shape of the tibial plateau and femorotibial contact mechanics after meniscectomy in the canine stifle

**DOI:** 10.3389/fvets.2025.1441329

**Published:** 2025-07-10

**Authors:** Antonio Pozzi, Pavlos Natsios, Stanley E. Kim, Christina J. Choate, Bryan P. Conrad

**Affiliations:** ^1^Department of Small Animal Clinical Sciences, University of Zurich, Zurich, Switzerland; ^2^Comparative Orthopaedics Biomechanics Laboratory, University of Florida, Gainesville, FL, United States; ^3^Grant Creek Veterinary Services, Missoula, MT, United States; ^4^Nike Sports Research Lab, Beaverton, OR, United States

**Keywords:** stifle, meniscectomy, contact mechanics, tibial plateau geometry, lateral meniscus, osteoarthritis

## Abstract

**Objectives:**

The purpose of this study was to describe the tibial plateau surface geometry and the contact mechanics after medial and lateral partial and total meniscectomies in the canine stifle.

**Study design:**

This was an *ex vivo* experimental study. Contact area, average contact pressure, and peak contact pressure (PCP) were recorded using a digital pressure sensor. The articular surfaces of each stifle’s tibia and femur were digitally mapped using a three-dimensional laser scanner.

**Results:**

Based on the normalized data, lateral total meniscectomy caused significantly greater changes in PCP than medial total meniscectomy. In contrast, partial meniscectomy, whether medial or lateral, did not result in any significant differences in PCP. With a total meniscectomy, mean PCP increased by 72 and 273% for the medial and lateral meniscus, respectively. Based on the absolute values, the PCP after lateral partial meniscectomy was significantly higher than after medial partial meniscectomy. The radius of curvature of the lateral tibial plateau (12.8 ± 10.9 mm) was smaller than the radius of the medial tibial plateau (25.9 ± 17.8 mm) (*p* < 0.05).

**Conclusion:**

Based on our results, the geometry of the medial and lateral tibial plateau may explain the different contact mechanics following medial and lateral meniscectomy. A total lateral meniscectomy may have greater detrimental effects when compared to medial meniscectomy.

## Introduction

The role of menisci as load-bearing elements has been studied in depth in human and animal models (1–6). Several studies have shown that meniscectomy causes a marked increase in femorotibial peak contact pressure, and that these changes in stress distribution cause detrimental remodeling of bone and soft tissue (4, 7, 8). Canine menisci transmit 65% of the joint reaction force and consequently meniscectomy causes a two-fold increase in compressive deformation of cartilage (8). In a canine cadaveric model, removal of the caudal horn of the medial meniscus caused a focal area of high pressure in the caudal region of the medial tibial condyle, corresponding to pressures higher than 10 MPa (9).

Custom-made piezoelectric pressure sensors have been used to measure the pressure and contact area (CA) of the canine stifle following varying degrees of meniscal resection (8, 10). These studies in dogs confirmed that total meniscectomy had a more detrimental effect on contact pressure when compared to an untreated meniscal tear (8). As reported for the human meniscus (4), the amount of meniscal tissue resected determined the magnitude of the increase in cartilage pressure (6). Small partial meniscectomies had minimal effect on meniscal function, whereas large partial and segmental meniscectomies resulted in significant changes in meniscal function (11).

Lateral partial and total meniscectomies seem to have a poor prognosis in dogs and in people (12–15). Krier et al. described severe articular cartilage lesions associated with lateral meniscal tears in 17 dogs (14). In a large prospective cohort study in people, lateral meniscus status had a significant influence on the long-term outcome, leading to the recommendation to treat most lateral meniscal tears conservatively (13). It has been suggested that lateral meniscectomy may be more detrimental than medial meniscectomy because of differences in joint congruity of medial and lateral tibial condyles (16). In humans, the medial tibial plateau is concave and on the lateral side is convex in the sagittal plane (17). This difference in surface geometry of the tibial condyles may play a role in the biomechanical effect of medial and lateral meniscectomy (16). However, no previous studies investigated the relationship between the contact pressures after meniscectomy and the geometry of the tibial plateau.

The dog is an established model to study the effect of meniscectomy both *in vivo* and *ex vivo* (5, 7, 11). Furthermore, the bony anatomy of the canine stifle is very similar to the human knee, with the convex-shaped lateral tibial plateau articulating with a convex-shaped lateral femoral condyle (17). The purpose of this study was to describe the changes in femorotibial CAs and pressures after partial and total meniscectomies of the medial and lateral menisci and the tibial plateau geometry of the canine stifle. The hypotheses were as follows: (1) lateral meniscectomy would result in a greater increase in contact pressures and a greater decrease in CA than medial meniscectomy; (2) the lateral tibial condyle would have a smaller radius of curvature than the medial tibial condyle.

## Materials and methods

### Specimen preparation and sensor placement

Ten pairs of hind limbs were harvested from large-breed canine cadavers euthanatized for reasons unrelated to this study, and approved by the relevant institution’s animal care and use committee (University of Florida IACUC #201106858). Hind limbs were harvested from the cadavers within 12 h following euthanasia. Radiographs of the tibiae were obtained to ensure skeletal maturity and the absence of joint pathology, as well as to measure the tibial plateau angle. All periarticular soft tissues were removed, while preserving the collateral and the cruciate ligaments. The portion of the femur proximal to the lesser trochanter, the distal tibial metaphysis, and the portion of the fibula 2 cm distal to the fibular head were osteotomized to allow placement within the testing apparatus. A 1.1 mm diameter Kirschner wire was drilled through the head of the fibula and proximal tibia, bent flush against the fibula, and trimmed 5 to 8 mm from the fibula to stabilize the fibular head and insertion of the lateral collateral ligament. The stifles were wrapped in physiologic saline-soaked towels and stored in a freezer at −20°C until they were thawed to room temperature for testing.

The potted specimens were thawed to room temperature while remaining wrapped in saline-soaked towels 1 h prior to mechanical testing. A stifle approach by osteotomy of the origin of the lateral collateral ligament was used to expose the medial and lateral compartments, insert the pressure sensors, and perform meniscectomies. A 3.5 mm diameter bone tunnel was drilled through the femoral condyle, centered at the origin of the lateral collateral ligament of the stifle. An osteotome was used to outline a block of bone that contained the entire origin of the lateral collateral ligament. The block of bone was freed from the underlying condyle using an osteotome allowing for the lateral collateral ligament to be reflected.

**Figure 1:**
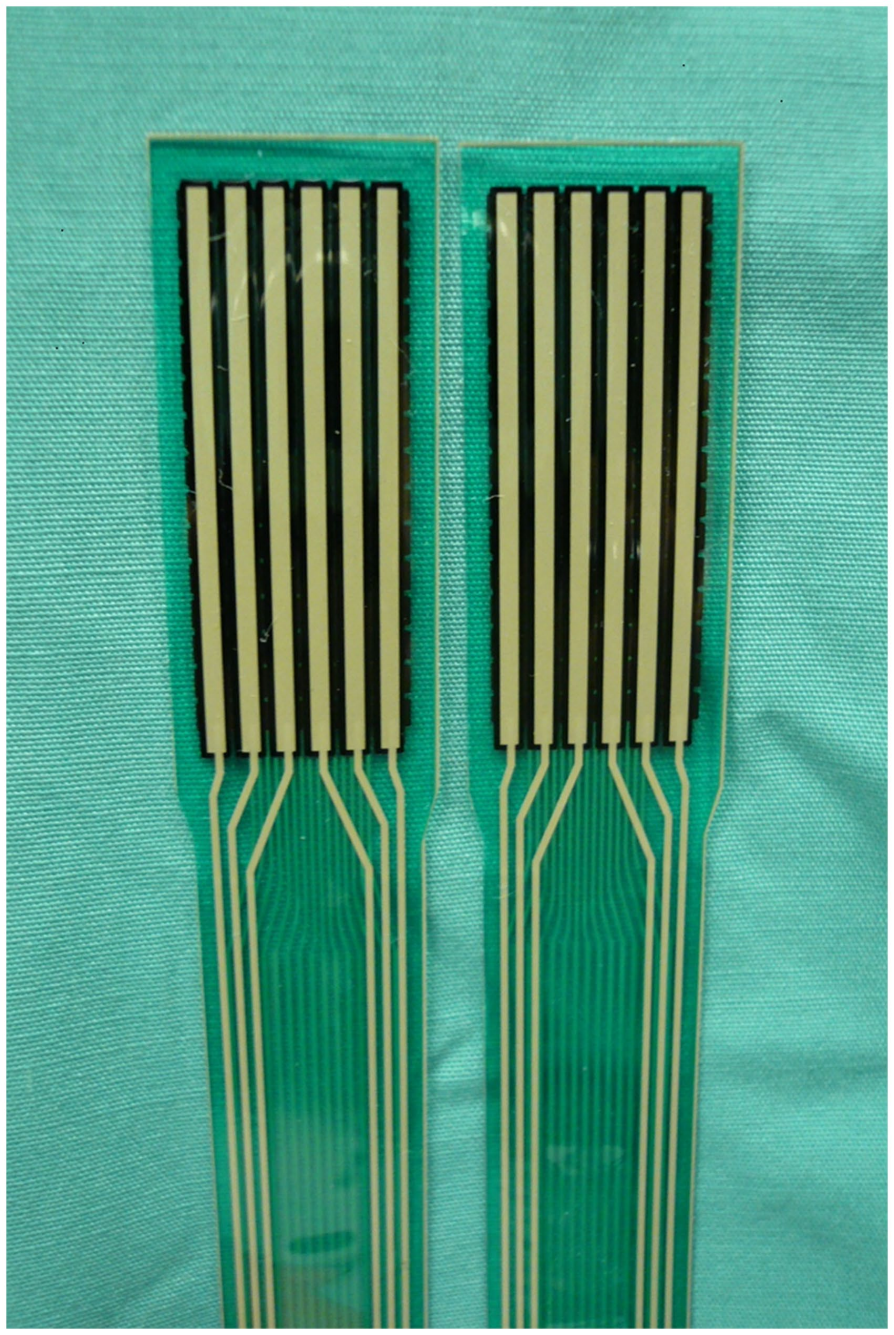
Piezo-resistive pressure sensors for the canine stifle (Tekscan Inc., South Boston, United States). The sensor had two separate sensing areas of 30.9 × 12.0 mm and a thickness of 0.08 mm. Each sensing area contains six rows and 15 columns of sensing elements providing 90 sensors. The sensors had a pressure sensitivity of 0.01 MPa and a pressure range of 0.5–30.0 MPa.

CA, peak contact pressure (PCP), and mean contact pressure (MCP) were recorded from a piezo-resistive pressure sensing system (Tekscan Inc., South Boston, United States). The sensor had two separate sensing areas of 30.9 × 12.0 mm and a thickness of 0.08 mm. Each sensing area contained six rows and 15 columns of sensing elements providing 90 sensors. The sensors had a pressure sensitivity of 0.01 MPa and a pressure range of 0.5–30.0 MPa. Each new sensor was conditioned then calibrated with a 10 mm diameter indenter with an applied force of 15 N as described by the manufacturer’s guidelines (Figure 1). The contact map was recorded during the calibration and the calibration curve was calculated based on a software program provided by the sensor manufacturer immediately prior to testing each specimen (Tekscan Inc., South Boston, United States).

The pressure sensors were inserted under each meniscus after exposing the relative compartment with flexion and rotation of the femoral condyles relative to the tibia. The sensors were advanced under each meniscus axially and cranially until the entire width was within the joint space. The sensors were secured to the tibial tuberosity with a 1.1 mm diameter olive wire inserted through the cranial margin of the sensors, which did not contain any sensing units. Sensor positioning was accomplished without causing any damage to the sensor by grasping it at its cranial and caudal edges. After positioning the sensor, the stifle was reduced, and the lateral collateral ligament osteotomy was fixed securely with a 3.5 mm screw, washer, and nut.

### Mechanical testing

A testing fixture was used to mount the limb to the materials-testing machine. The stifle was positioned at 135 degrees of flexion, measured using a goniometer. To allow even distribution of compartmental forces, the stifle was loaded to 100 N axial load and the varus/valgus alignment and medio-lateral translation were manually adjusted until the load was distributed evenly between the medial and lateral compartments. Then the rotational and medial/lateral translational movements of the jig were locked to ensure that the specimen would be loaded axially with two degrees of freedom (antero-posterior translation and axial rotation).

Mechanical testing was performed using an axial servohydraulic dynamic mechanical testing machine (858 MiniBionix II, MTS Systems Corp, Eden Prairie, MN). An axial force of 100 N was applied to each specimen over 5 s and then maintained for 11 s. A force of 100 N was chosen because it represents the force of about 50% of the body weight of a 20-kg dog, based on a previous kinetic study in normal dogs (18).

Contact maps that allowed determination of instantaneous CA, MCP, and PCP were recorded from each stifle in each of the tested conditions. Contact maps were recorded 15 s into the loading protocol. The measurements were detected using a piezo-resistive pressure sensing system (Tekscan Inc., South Boston, United States).

### Surgical procedures

Contact maps were recorded in each stifle for three different conditions: control (intact meniscus), 50% partial meniscectomy (removal of the axial part of the meniscus, preserving the caudal and cranial meniscal ligaments), and total meniscectomy. CA, MCP, and PCP measurements were recorded. Surgical procedures were performed sequentially after testing each condition. The control condition was tested with both menisci intact. One stifle from each pair then underwent serial meniscectomy of the medial meniscus, and the paired stifle underwent a serial lateral meniscectomy. After a contact map was recorded with the meniscus intact, the femur was removed from the jig, and the screw securing the collateral attachments was removed. The femur was then rotated externally to allow access to the lateral meniscus or rotated internally to allow access to the medial meniscus. The width of the meniscus was measured at the level of the ipsilateral collateral ligament and 50% of the meniscus was excised using a #11 blade. The femur was reduced, the femoral collateral ligament attachment was secured with the screw, and the femur was inserted in the jig in the original position to maintain the same orientation. After recording a contact map for the partial meniscectomy condition, the meniscus was removed, and another contact map was recorded.

### Measurement of tibial plateau geometry

A three-dimensional (3D) laser scanner (3D Scanner HD, NextEngine, Santa Monica, CA) was used to scan the tibiae. From the 3D images, a series of 15 to 20 points located in the sagittal planes bisecting the medial and lateral tibial plateau, approximately 1 to 2 mm apart, was digitized along the surface of the medial and lateral tibial plateau. These points were used to fit a sphere to the bone curvature using a least-squares optimization routine in MATLAB (R2016a, MathWorks, Natick, MA). The calculation produced the radius of the sphere that best fit the point data for both the medial and lateral tibial plateau.

### Data analysis

Statistical analysis was performed using a commercially available software system (Prism 5, GraphPad Software, Inc., La Jolla, CA). A two-way repeated measures ANOVA was used to compare the absolute values of CA, MCP, and PCP following a 50% partial and total medial and lateral meniscectomy. The effects of 50% partial and total meniscectomy, calculated as percent increase or decrease from the control to account for the anatomical differences among specimens were compared between the medial and lateral meniscus using a Mann- Whitney test. For all statistical analyses, significance was set at *p* < 0.05. A sample size calculation based on previously published data was performed (alpha 0.05 and 90% power), showing that a sample size of *n* = 4 per group was sufficient to detect a difference.

## Results

Both lateral and medial total meniscectomy resulted in an increase in PCP of 273 and 72%, in MCP of 309 and 109%, and a decrease in CA of 66 and 57%, respectively (Tables 1, 2). Based on the absolute values, there were statistically significant decreases in CA values after total meniscectomy for the lateral meniscus and after partial meniscectomy for the medial meniscus (*p* < 0.05). For total medial meniscectomy and partial and total lateral meniscectomy groups, PCP was significantly increased compared to controls (*p* < 0.05). Both partial and total meniscectomies caused statistically significant increases in MCP for both medial and lateral menisci (*p* < 0.05). When comparing the changes in CA, PCP, and MCP between medial and lateral meniscectomy groups, only the PCP increases after partial meniscectomy were significantly different between the medial and lateral menisci (Table 1).

**Table 1:** Contact mechanics absolute data (mean ± SD) for the normal, partial meniscectomy and total meniscectomy conditions.

Variable		Normal (1)	Partial (2)	Total (3)	*p*-value
CA (mm^2^)	Medial	81.1 ± 22.6	62.5 ± 17.5	34.3 ± 14	*P*_1-2_ < 0.05
	Lateral	84.5 ± 27.3	64.4 ± 20.7	24 ± 5.3	*P*_1-3_ < 0.001
p-value (med vs. lateral)		NS	NS	NS	
PCP (MPa)	Medial	1.4 ± 0.5	1.8 ± 0.7	2.3 ± 0.9	*P*_1-3_ < 0.05
	Lateral	1.2 ± 0.4	1.2 ± 0.4	2.7 ± 0.7	*P*_1-3_ < 0.05 *P*_2-3_ < 0.001
p-value (med vs. lateral)		NS	0.02	NS	
MCP (MPa)	Medial	0.8 ± 0.4	0.9 ± 0.4	1.4 ± 0.5	*P*_1-3_ < 0.001 *P*_2-3_ < 0.01
	Lateral	0.6 ± 0.5	0.7 ± 0.5	1.4 ± 0.3	*P*_1-3_ < 0.001 *P*_2-3_ < 0.001
p-value (med vs. lateral)		NS	NS	NS	

CA, contact area; PCP, peak contact pressure; MCP, mean contact pressure; NS, not significant. *p*-values for post-hoc pairwise comparisons listed in the right column are given where significant differences were detected by ANOVA. *p*-values listed in the right column are given where significant differences were detected by ANOVA. *P*-values listed in the rows below each variable are given where significant differences were detected by paired *t*-test when comparing variables between medial and lateral compartments.

**Table 2:** Contact mechanics normalized data presented as percentage decreases in CA, and increases in PCP, and MCP after partial and total meniscectomy conditions.

Variable		Partial	Total
CA (mm^2^)	Medial	21%	57%
	Lateral	22%	66%
*P*-value (medial vs. lateral)		0.9	0.1
PCP (MPa)	Medial	30%	72%
	Lateral	30%	273%
*P*-value (medial vs. lateral)		0.9	0.02
MCP (MPa)	Medial	16%	109%
	Lateral	20%	309%
P-value (medial vs. lateral)		1	0.3

CA, contact area; PCP, peak contact pressure; MCP, mean contact pressure. *P*-values listed in the rows below each variable are given for each comparison between medial and lateral compartments using a Mann–Whitney test.

Based on the analysis of the normalized data (Table 2), there was a significance difference in PCP after total meniscectomy between medial and lateral meniscus (*p* < 0.05).

**Figure 2:**
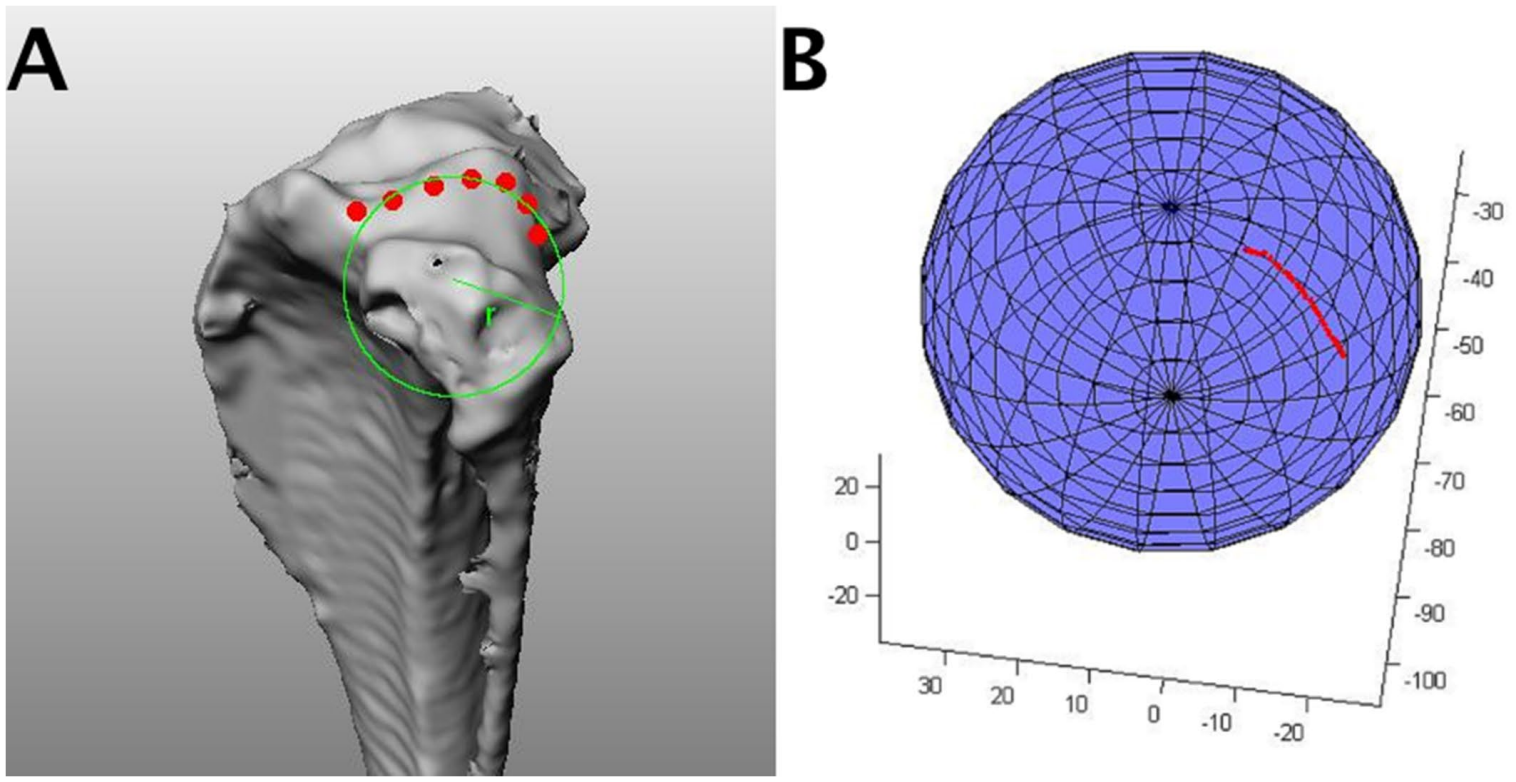
An example of a sphere fit algorithm. **(A)** From the 3D scans of the tibia, a series of 15–20 points lying in the sagittal plane approximately 1–2 mm apart was digitized along the surface and in the center of the medial and lateral tibial plateau. **(B)** These points were used to fit a sphere to the bone curvature using a least-squares optimization routine in Matlab (SphereFit, Mathworks, Natick, MA). The calculation produced the radius of the sphere that best fit the point data for both the medial and lateral tibial plateau.

The radius of curvature of the lateral condyle (mean ± SD: 12.8 ± 10.9) was significantly smaller than the radius of curvature of the medial condyle (mean ± SD: 25.9 ± 17.8) (*p* < 0.05) (Figure 2).

## Discussion

The primary goal of the study was to test the hypothesis that tibial plateau geometry correlates with the post-meniscectomy contact mechanics in the canine stifle. Based on our results, differences in surface geometry between the medial and lateral tibial plateau may be responsible for the differences between lateral and medial contact mechanics following meniscectomy. The radius of curvature of the lateral condyle was significantly smaller than the radius of the medial tibial condyle, supporting the theory that the convexity of the lateral tibial plateau induces a greater magnitude of increased PCP and decreased CA, due to focal loading following lateral meniscectomy (16).

The combination of loss of meniscal function and tibial plateau geometry may explain the fast progression of OA in the lateral compartment (14). Several studies of the human and canine femorotibial joint indicate that lateral meniscectomy causes greater disruption than medial meniscectomy (12, 13, 19, 20). A 3D finite element study of the human femorotibial joint showed that the peak contact stress and maximum shear stress in the articular cartilage increased 200% more after a lateral than a medial meniscectomy (17). Our contact mechanics data demonstrated that a similar pattern occurs in the dog, although not as severe as the changes observed in the human knee. The greater effect of lateral meniscectomy in people may be attributed to the more pronounced asymmetry of the medial and lateral tibial condyles (21). The canine tibial plateau is convex in both canine tibial condyles, while the medial tibial condyle in people is slightly concave (21).

We found that the radius of curvature of the lateral tibial plateau was 50% of the medial plateau. The geometry of the lateral and medial tibial plateau has been already described by Townsend et al. (22) Loss of the lateral meniscus from the convex articular surface of the lateral tibial plateau may not only influence contact mechanics but could also affect joint stability. Given the greater convexity of the tibial articulating surface, the lateral compartment may be inherently more susceptible to anterior–posterior shear motion than the medial compartment. Loss of the lateral meniscus may predispose to rotational instability, a problem increasingly recognized in dogs (23–26). The importance of the articular surface geometry in knee joint stability has been reported for human patients with anterior cruciate ligament injury (27). Athletes with a non-contact anterior cruciate ligament injury were found to have a smaller and more convex lateral tibial plateau, leading to the conclusion that this anatomical feature may be a predisposing factor for rupture (28). When considering the convexity of both tibial and femoral articular surfaces in the lateral compartment, it is likely that the lateral meniscus plays a crucial role in congruity and stability of the lateral stifle compartment. Removal of the lateral meniscus increases anterior tibial translation during combined valgus and rotatory loads, further supporting a conservative approach to treatment of lateral meniscal injuries (29).

Our study confirms that tibial plateau geometry affects biomechanics as suggested by several studies in dogs and people (22, 30). Despite the different methodology, the results are similar to Townsend et al. that reported the medial tibial plateau radius in dogs with and without cranial cruciate ligament rupture (22). These authors used reconstructed computed tomography images, a method that can be applied to any clinical patient. Our data and Townsend et al. results suggest that a calculation of the radius of curvature rather than an estimation of the tibial plateau may be considered as part of the diagnostic work-up in complex stifle cases.

Some limitations of the contact pressure and area measurements should be considered when interpreting our results. The custom-designed Tekscan sensor may alter the measured pressure because it is stiffer than articular or meniscal cartilage (31). An additional source of error is sensor discretization, which ranges between 1 and 9% for pressure and areas, but may be higher in small CA (32). Beyond the limitations of the method of measuring contact mechanics, the setup and design of the study should be considered. *In vivo* contact mechanics are affected by complex joint surface interactions that may not be reflected in our static model (33). Another limitation of the methodology is that the capsular attachments of the menisci were transected to allow sensor placement. This specimen preparation is necessary to place the sensor precisely to cover the whole tibial plateau (5, 11), but alter the normal soft tissue attachments of the meniscus. Additionally, the impact of the surrounding musculature that contribute to stifle biomechanics cannot be evaluated in this study due to the need for their removal for the set up.

In conclusion, the consequences of meniscectomy may depend on the surface geometry of the knee compartments (16). The lateral meniscus plays a major role in improving the congruity of the convex lateral femoral and tibial condyles by covering a larger proportion of the compartment (34). The crucial role of the lateral meniscus in joint congruity, load transmission, and joint stability should be considered when treating lateral meniscal injuries. Based on our data we support a conservative treatment approach by avoiding resection of stable medial and lateral meniscal tears. Preserving as much lateral meniscal tissue as possible may improve lateral compartment contact mechanics.

## Data Availability

The raw data supporting the conclusions of this article will be made available by the authors, without undue reservation.
